# Supplementary material for: Identification, Characterization, and Stress Responsiveness of Glucose-6-phosphate Dehydrogenase Genes in Highland Barley
Source: Plants (Basel). 2020 Dec 18;9(12):1800. doi: 10.3390/plants9121800 (PMC7766724; doi:10.3390/plants9121800)
Supplement: Supplementary file 1 [file plants-09-01800-s001.pdf]

Table S1. Primers used in this study.

Primer name	Primer sequence (5′ to 3′)	Purpose
HvG6PDH1-F	CGCCACGAGACTAGAAAGAAGC	Full-length cDNA amplification for *HvG6PDHs*
HvG6PDH1-R	TTGGGATGGAGGGAGTAGTAGCT	
HvG6PDH2-F	CCAGCATCCCAGCAGTAACTCT-3′	
HvG6PDH2-R	CATATTAGGTCGCCTTACCGTCG-3′	
HvG6PDH3-F	AACCCCAATCCAGCACCAAAG	
HvG6PDH3-R	ACCAACAGCTTTTGCAGTTACAC	
HvG6PDH4-F	CACCATTCGTCAGAGCCAAGCAT	
HvG6PDH4-R	AGTAGAGTGCATAGGCCGTAGCC	
HvG6PDH5-F	GGACGGAGAAACCATACAGAC	
HvG6PDH5-R	CTCTGTAAACCCAGAACACTC	
HvG6PDH1-P-F	CATGCCATGGCGCTCTCCTGCATGA	Link of *HvG6PDHs* CDS and PET28(a)
HvG6PDH1-P-R	CCGCTCGAGGCAGCCATCATCCCATCGGA	
HvG6PDH2-P-F	CATGCCATGGCGGGAACTGACTCCTC	
HvG6PDH2-P-R	CCGCTCGAGTGCAAGGGTGGGTGGTATCC	
HvG6PDH3-P-F	CATGCCATGGCGCTCTCCTGCATGA	
HvG6PDH3-P-R	CCGCTCGAGGTGTTCTGATCCTCCTAGGT	
HvG6PDH4-P-F	GGGAATTCCATATGATGGCGCTCTCCTGCATG A	
HvG6PDH4-P-R	ACGCGTCGAC CAAGGATCCGTCGCTGCTGAT	
HvG6PDH5-P-F	CGGATCCATGTCAGGAGGATCGGGTGT	
HvG6PDH5-P-R	CCGCTCGAGAAACTTCGCAAGGGTTGGTG	
HvG6PDH1-L-F	CACCATGGTTACGACAGTCCTCGC	Localization for *HvG6PDHs*
HvG6PDH1-L-R	GCATCCATCATCCCATCTGACT	
HvG6PDH2-L-F	CACCATGGCGGGAACTGACTCCTC	
HvG6PDH2-L-R	TGCAAGTGTGGGTGGTATCCAGA	
HvG6PDH3-L-F	CACCATGGCGCTCTCCTGCATGA	
HvG6PDH3-L-R	GTGTTCCGATCCTCCCAGGT	
HvG6PDH4-L-F	CACCATGGCCGCCATCGACTTA	
HvG6PDH4-L-R	CAAGGATCCGTCGCTGCTGATGT	

HvG6PDH5-L-F	CACCATGTCAGGAGGATCGGGT	
HvG6PDH5-L-R	AAACTTCGCAAGGGTTGGTG	
HvG6PDH1-R-F	TGAAACGGCAAATACAAGCTCT	Real-time PCR for *HvG6PDHs*
HvG6PDH1-R-R	ACTTTACTTCTTGCCAGTT	
HvG6PDH2-R-F	GAACCCGATAAAGGACGAAGA	
HvG6PDH2-R-R	TCTTGAGTTTAATGCTTTACC	
HvG6PDH3-R-F	AGCTACGGTATTATCCGAGACAT	
HvG6PDH3-R-R	ATGTAATACCACCCTTCGT	
HvG6PDH4-R-F	GTCCAATCCAAGCACCAAAC	
HvG6PDH4-R-R	TGATGCTGACGGTGTTCCCT	
HvG6PDH5-R-F	GTTGTACTAGGTGCTTCTGG	
HvG6PDH5-R-R	ACAATCTGTACGTTATCAA	
HvACTIN-F	GTGGTCGTACAACAGGTATT	Real-time PCR for *HvACTIN*
HvACTIN-R	TCTGTCAGGATCTTCATT	

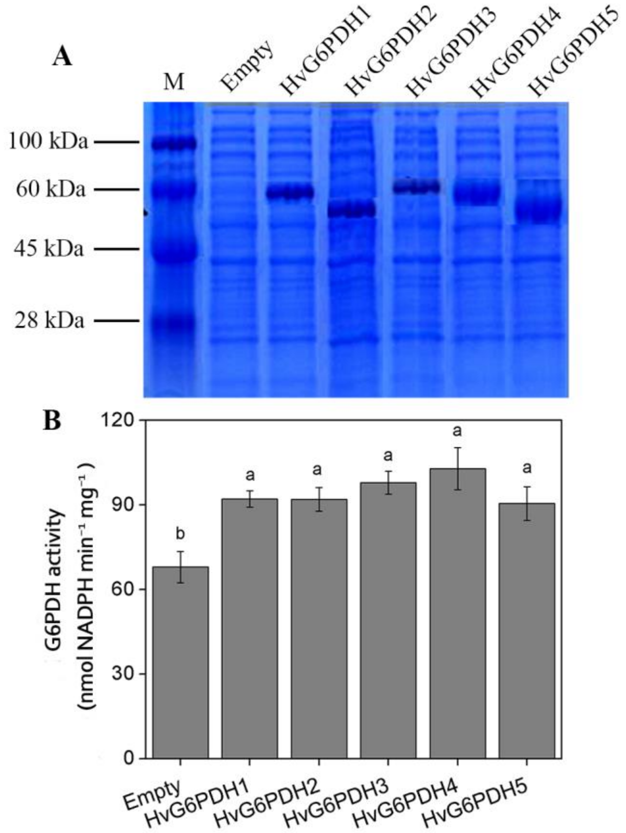

Figure S1. Expression of the five *HvG6PDH* genes and their enzyme activity in *E. coli*. (**A**) SDS-PAGE profile of protein extracts from ITPG-induced *E. coli*. The arrowheads showed the bands of HvG6PDH proteins. (**B**) The enzyme activity of G6PDH proteins in *E. coli*. The *E. coli* was transformed with pET28(a) empty and pET28(a)-HvG6PDHs (HvG6PDH1 to 5) vectors, respectively. Statistical

differences were analyzed on the basis of the Student's t-test, and bars with different letters were different at the 0.05 level.

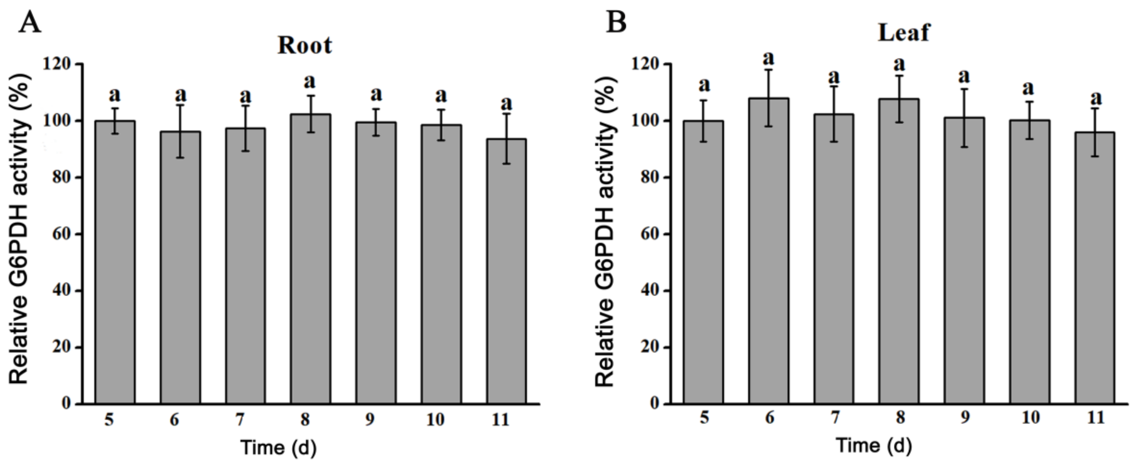

Figure S2. The analysis of total G6PDH activity in the leaves (**A**) and roots (**B**) of highland barley seedlings at the different growth stages. The highland barley seedlings were cultured in the 1/4-strength Hoagland media. Statistical differences between the G6PDH activity of 5-days-old seedlings and G6PDH activity at other time point were analyzed on the basis of Student's t-test, and bars with different letters were different at the 0.05 level.

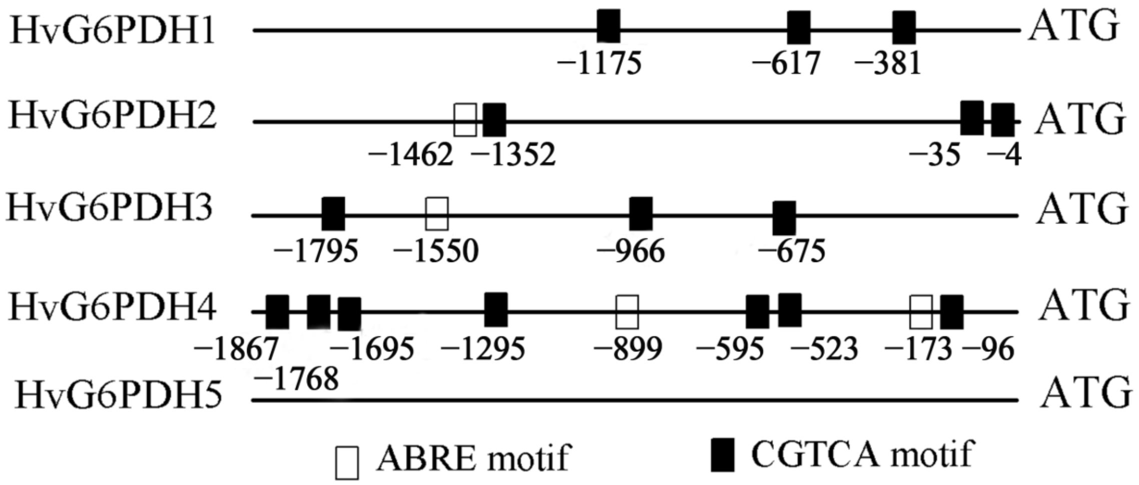

Figure S3. ABRE (white box) and CGTCA (black box) motifs in the promoter sequences (2000 bp) before initiation codon ATG of *HvG6PDH1-5* genes. ABRE and CGTCA motifs indicated the ABA and JA response elements, respectively. These motifs were predicted by online software plantCARE (http://bioinformatics.psb.ugent.be/webtools/plantcare/html/).

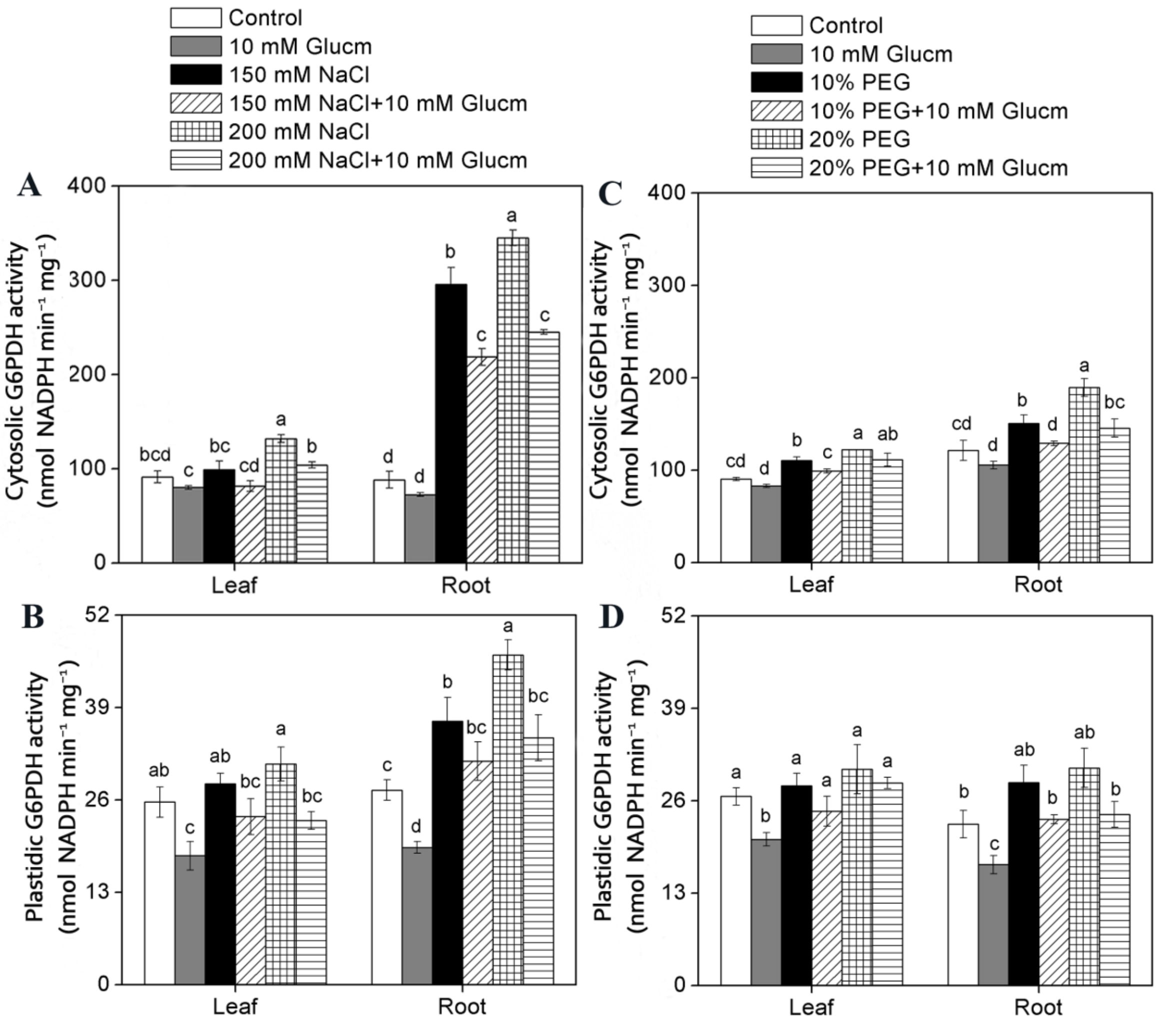

Figure S4. Effect of G6PDH inhibitor (Glucm) on the activities of cytosolic and plastidic G6PDH in leaf and root under NaCl (**A**,**B**) and PEG (**C**,**D**) stresses for 48 h from highland barley. Data are mean ± *SE* (n = 3). Statistical differences were analyzed on the basis of Student's t-test, and bars with different letters were different at the 0.05 level.
